# Association of age and gender of patients undergoing class V tooth coloured restoration in maxillary teeth

**DOI:** 10.6026/973206300161121

**Published:** 2020-12-31

**Authors:** Shreya Kothari, Manish Ranjan, Balaji Ganesh

**Affiliations:** 1 Saveetha Dental College, Saveetha Institute of Medical and Technical Sciences, Saveetha University, Chennai 77, India

## Abstract

It is of interest to assess the association of age and gender of patients undergoing class V tooth colored restoration in maxillary teeth. Records were collected by reviewing the data of 86,000 patients of which 1580 patients had undergone class V tooth colored restoration in maxillary teeth. Patients were divided into age groups 18-30, 31-40,41-50,71-80 years. The most common age group who had undergone class V restoration was in the age group 41-50 years (30.8%). Cervical abrasions were the most common (79%). Most of the patients underwent direct restoration (87.7%). Direct restoration was found to be more prevalent in the age group 41-50 years due to cervical abrasion.

## Background

Cervical lesions are lesions, which involve the gingival one-third of the facial and lingual surfaces of tooth crown [1]. These can be associated with caries or could be non carious in nature [2]. Regulation of such physiological or pathological conditions is carried out by different protease systems like MMPs, which produce odontoblasts and play a role in dental caries [3]. Lesions associated with caries are also termed as root caries, which could be primary or secondary. Primary caries occur due to absence of a restoration whereas secondary adjacent to an existing restoration. It is found that root caries are associated with age and gender with age and recession of gingiva. It is the region where plaque accumulation often occurs [4]. Enamel is found to undergo cycles of demineralisation and remineralisation throughout lifetime, which control the progression of caries [5 - check with author]. Root caries are found to be more prevalent in elderly people and most commonly seen in the facial or buccal surfaces of premolars and molars [6]. This is the reason that tooth colored restoration is chosen. The common materials of choice are Resin Composite or GIC [7]. Non-carious lesions lead to pathological loss of tooth tissue. It is either in the form of abrasive damage caused by brushing or erosion due to acid of non-bacterial origin. Sometimes, increased biomechanical loading forces can lead to a wedge shaped defect termed as abfraction [8]. It is suggested by some authors that pathological destruction of dental tissue is attributed to a non-carious process for about 25% [7]. The common area affected is the vestibular region. The enamel in these areas is found to be irregularly structured, gets thinner with a weak bond with the dentine [8]. The lesions are generally seen as shallow depressions, broad disk-shaped to large wedge shaped defects, which have a flat or indented floor [9]. The lesions also exhibit mixed cavity margins making it difficult to restore since the bond strength between enamel and dentine and with the material of restoration is affected [10]. It has become necessary to prevent further destruction or progression of these lesions getting more severe leading to the loss of vitality of the pulp [11 - check with author],[12]. This is achieved by choosing the right material for restoration. This is important to eliminate the sensitivity, carious progression and to enhance aesthetics [13]. Resin Composite has been the material of choice for a long time because of its versatility, high wear resistance, superior esthetics and has adequate strength [14]. But it is disadvantageous for reasons like technical difficulties like isolation, polymerisation shrinkage and adhesion to dentine margin [15]. Polymerisation shrinkage problems leading to debonding of the restoration can be dealt with by usage of flowable composites [16]. Another common material used is the Glass Ionomer Cement (GIC). It forms a chemical bond with enamel and dentine and has the property of releasing fluorides for a caries protective effect [17,18]. This material is inferior to composites when esthetics is the prime concern. Other teeth colored materials are the recent advances like Resin Modified GIC and polyacid modified resin composite. They are suggested to have less microleakage; stronger bond with mineralised tissue, no postoperative sensitivity and easier clinical handling [19-21]. Some clinical studies have proven that these two materials can be used for esthetic restoration of cervical lesions [22] and have good marginal adaptation as well [16] Survival of restorations depends not only on the type of material used. It depends on various other factors like isolation, operator's skill, patient compliance, oral environment and technique used [23]. Previously our team had conducted numerous clinical trials [24], lab animal and invitro studies [5, 11,^25^-^27^] and reviews [12,21,28-31- check with author] over the past 5 years. Now we are focussing on epidemiological surveys. Therefore, it is of interest to assess the association of age and gender of patients undergoing class V tooth coloured restoration in maxillary teeth.

## Materials and Methodology:

### Study setting:

This was a retrospective study of patients who had undergone tooth coloured class V restoration in maxillary teeth. It revolved around a university setting having patients visiting a private dental college and hospital in between June 2019- April 2020. The approval for this study was taken from the ethical committee with the approval number SDC/SIHEC/2020/DIASDATA/0619-0320. The sample size of this study was 1580 patients. Verifying the photographs minimized sampling bias. Inclusion criteria were all patients who had tooth coloured class V restoration done in maxillary teeth. Inclusion criteria were all adult patients who had any class V restoration in maxillary teeth. Exclusion criteria for this study were all the patients who had class V restoration done in mandibular teeth.

### Data Collection and Tabulation:

We reviewed the patient records and analyzed the data of 86,000 patients. After collecting the data, it was tabulated. Tabulation included information/ parameters - Name of the patient, Age, Gender, Diagnosis and type of restoration. Age was categorized into 4 groups - 18-30, 31-40, 51-60, 61-70, 71-80. The diagnosis was an abrasion/caries/abfraction. Type of restoration was either Direct/ Bi-layered restoration.

### Statistical Analysis:

After further verification of data by an external reviewer, IBM imported it to the SPSS software for statistical analysis. Percentages, mean, Frequency of certain parameters were employed in the analysis. Chi-square test was used to detect the significance between age/gender with type of restoration and age & gender association too. p value <0.05 was considered to be statistically significant.

## Result:

The data collected and imported to SPSS software was used for descriptive statistics. The total sample size of the patients was 1580. It was seen from Figure 1(see PDF) that there was a higher prevalence of Males (71.0 %) who have undergone Class V restoration as compared to Females (28.9 %). Figure 2 (see PDF) shows the prevalence of different age groups and it was found that the maximum number of patients was present in the age group 41-50(30.8%) and the least in 71-80 years (2.4%). Diagnosis revealed a higher percentage of cervical abrasions (79.5%) and lesser of caries (19.4%) as seen in Figure 3 (see PDF). Figure 4 (see PDF) shows that the restoration was either direct or bi-layered (composite and GIC). 87.7% of the restorations were directly restored and 12.2% was bi-layered. Chi square tests were performed to find the association of age and gender and its association with type of restoration individually. Figure 5(see PDF) shows the association of age with type of restoration and reveals that both Direct and bilayered restoration are more common in 41-50 years of age group than in 51-60 years. Association of gender and type of restoration in Figure 6(see PDF) shows direct restoration is the most common in male and females. Figure 7(see PDF) show that both males and females of the 41-50 years age group show higher prevalence than patients above 50 years. There is a decrease in both males and females in the prevalence of restoration from 41-50 years to 51-60 years age group. All the three chi square tests proved to be insignificant( p value < 0.05).

## Discussion:

The above results show that the most common age group who has undergone class V restoration is 41-50 years (30.8 %) followed by the 51-60 year age group (25.8%). This was in concordance with a previous study done which says that the frequency of cervical lesions increased in a similar age group by 10 % as compared to other age groups [32]. The number of cervical lesions had significantly decreased in the age group 61-70 years by 12%. A study done by Husna et al also showed a decrease in the prevalence in people above the 65 years age group [33]. Meanwhile, gender analysis shows that Males are more prevalent (71%) than females (28.9%). There is a significant difference in the incidence of gender. A study done by Aw et al does not support this finding and says that there is no difference in the incidence of cervical lesions in males and females [34]. Our study showed that there is a higher rate of occurrence and presence of cervical abrasions (79.5%) than caries and abfraction. This was similar to a previous study done where there was an incidence of 71% of lesions found to be abrasions [35]. The reason why Abrasion is more common is because of the improper method of brushing, which leads to increased tooth wear. Also, the enamel here is aprismatic, which is resistant to caries because of less solubility in acid thereby showing high prevalence of Non-Carious Cervical Lesion [36]. But the tooth wear can lead to progressive softening of the enamel and dentin surface due to increased acid attack causing dissolution of the prism cores and interprismatic areas in prismatic enamel [27]. Patients often complain of sensitivity due to exposure of the dentine surfaces in cervical regions, which can be managed with desensitising pastes. Chlorhexidene has been proved to have various uses and shows that the application of 2% Chlorhexidine solution after restoration helps in reducing the sensitivity in posterior teeth [29,37,38]. Restoration of these cervical lesions was either direct or bilayered. Direct restoration was done in 87.7% of patients and it was bilayered in 12.2%. In support of our study, another study also had 80% usage of Resin composite, which is direct restoration [39]. Various types of composites are available nowadays for these restorative procedures wherein microfill composites have high degree of smoothness as compared to hybrid composites, which are less esthetic [40]. Association between age and type of restoration shows that both the types of restoration were common in 41-50 years of age group. This was contradicted by a study done by Kolak et al who said that percentages of multiple lesions and restorations increased in the age group of 55 years and above [8]. Direct restoration is more common than bilayered in both males and females. Association of age and gender reveals that both males and females are more prone to cervical lesions and underwent restorative treatment in the group 41-50 years. It also shows that there is a decrease in the prevalence of class V restoration in patients of age between 51-60 years compared to 41-50 years. This association proves to be insignificant (p value > 0.05). The study has its limitations because it studies a small size of population and iis single centred. It cannot be generalised as it gives information regarding class V restorations pertaining only to maxillary teeth. Therefore we should encourage an extensive research, which involves a large population further increasing the scope for more in future. It will bring awareness to the people and improve the stability of cervical regions of the tooth by development of different ways to treat the same.

## Conclusion

Within the limitations of the study, we can conclude by saying that the most common age group prevalent in class V restorations is 41-50 years age group and is higher in males. Direct restoration is more common than bi-layered restoration in both males and females. The most common cause for the treatment of class V restoration was found to be cervical abrasions.
